# Supplementary material for: A membrane associated tandem kinase from wild emmer wheat confers broad-spectrum resistance to powdery mildew
Source: Nat Commun. 2024 Apr 10;15:3124. doi: 10.1038/s41467-024-47497-w (PMC11006675; doi:10.1038/s41467-024-47497-w)
Supplement: Supplementary file 1 — Supplementary information [file 41467_2024_47497_MOESM1_ESM.pdf]

A membrane associated tandem kinase from wild emmer wheat confers broad-spectrum resistance to powdery mildew

Li *et al*.

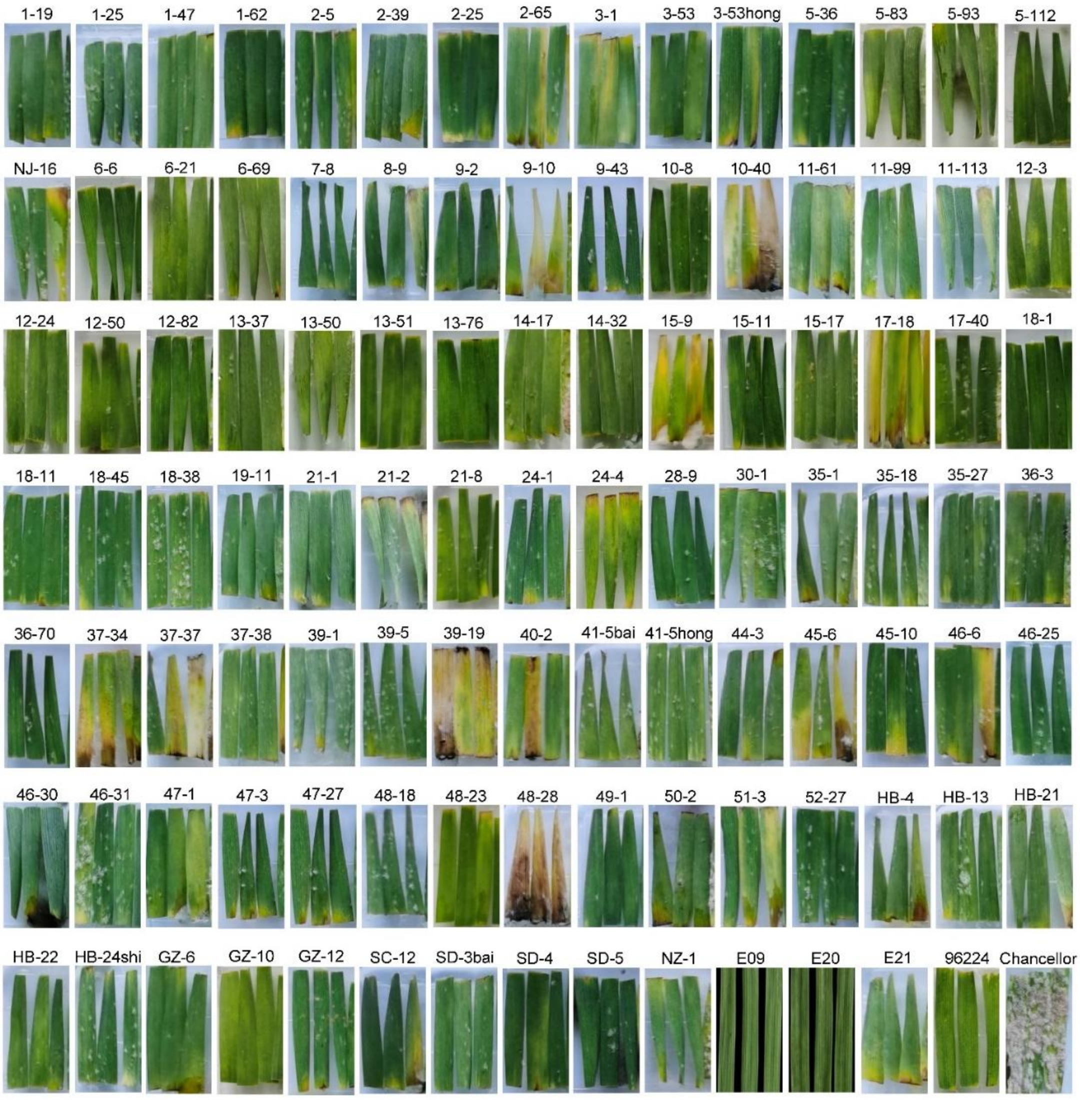

Supplementary Fig. 1. Infection reactions of line 3D232 to *Bgt* isolates. Common wheat cultivar Chancellor was used as the susceptible control. Detached leaves from seedlings of 3D232 and Chancellor at two-leaf stage were inoculated with 104 tested *Bgt* isolates, respectively. Representative leaves were photographed at 10 days post inoculation (dpi). Source data are provided as a Source Data file.

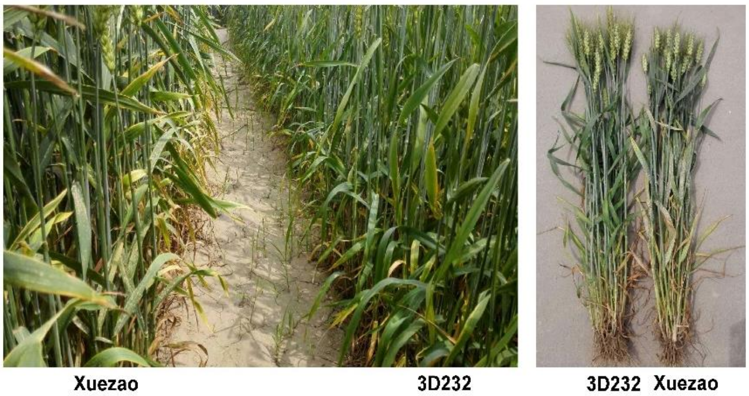

Supplementary Fig. 2. Infection reaction of line 3D232 to *Bgt* isolate E09 at the adult plant stage. Lines 3D232 and Xuezao at jointing stage were inoculated with *Bgt* isolate E09 under field condition. Powdery mildew reactions were evaluated at the grain-filling stages. Line Xuezao was also used as the susceptible control. Source data are provided as a Source Data file.

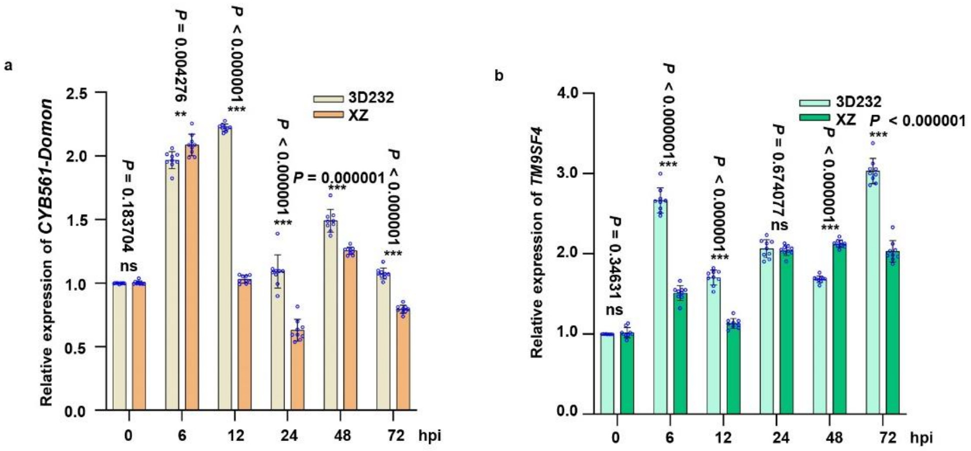

Supplementary Fig. 3. Genes expression analysis in the *Ml3D232* genetic region. Relative expression level of *CYB561-Domon* (**a**) and *TM9SF4* (**b**) were examined by quantitative reverse transcription PCR (qRT-PCR). Leaves of 3D232 and Xuezao seedling plants inoculated with *Bgt* isolate E09 at the two-leaf stage were collected at 0, 6, 12, 24, 48, and 72 hour post inoculation (hpi). *TaActin* was used as an endogenous control. Data are means ± SD from three biological replicates (three leaves used per biological replicate) and three technical replicates for each leave were performed (n=9). Statistical significance was determined using unpaired two-tailed *t* test. ns, not significant; **, P<0.01; ***, P<0.001. Source data are provided as a Source Data file.

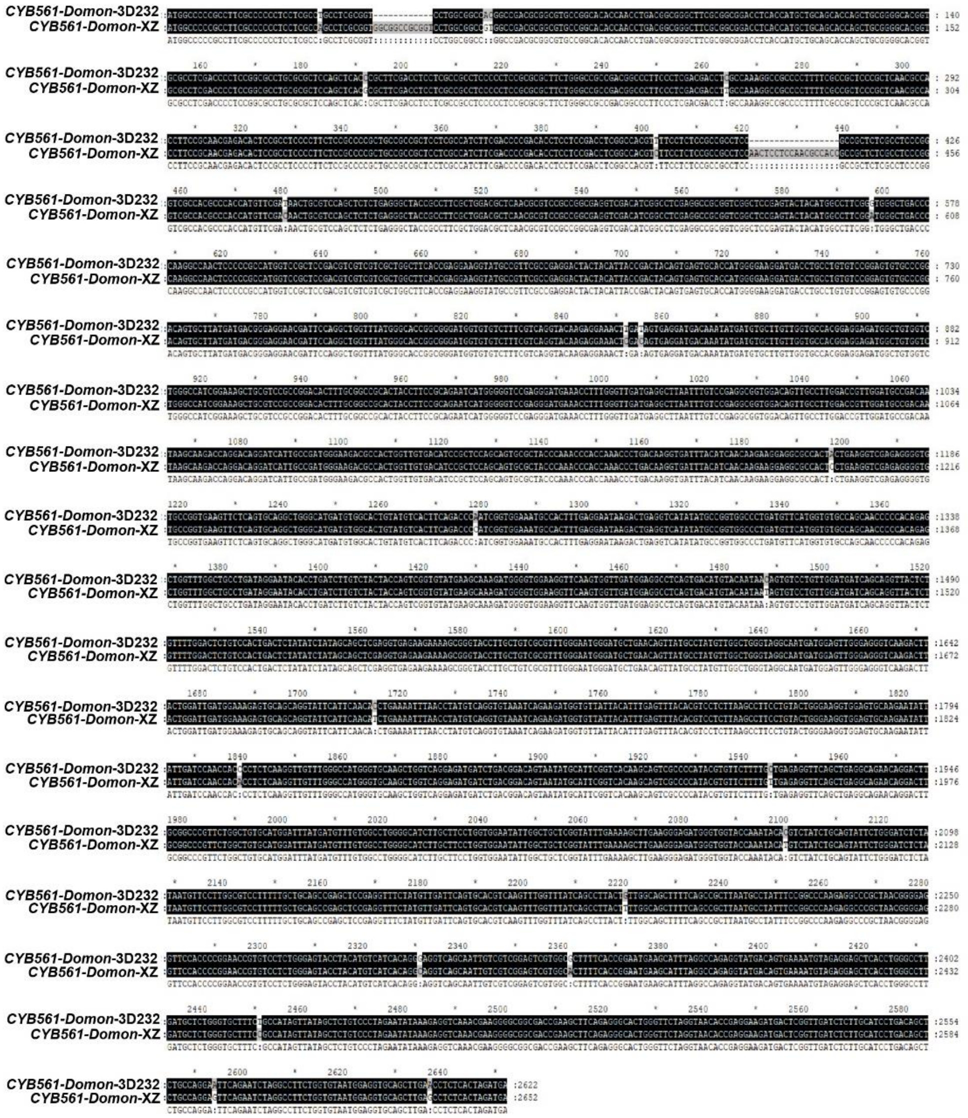

Supplementary Fig. 4. Coding sequence alignment of *CYB561-Domon* gene from lines 3D232 and Xuezao (XZ).

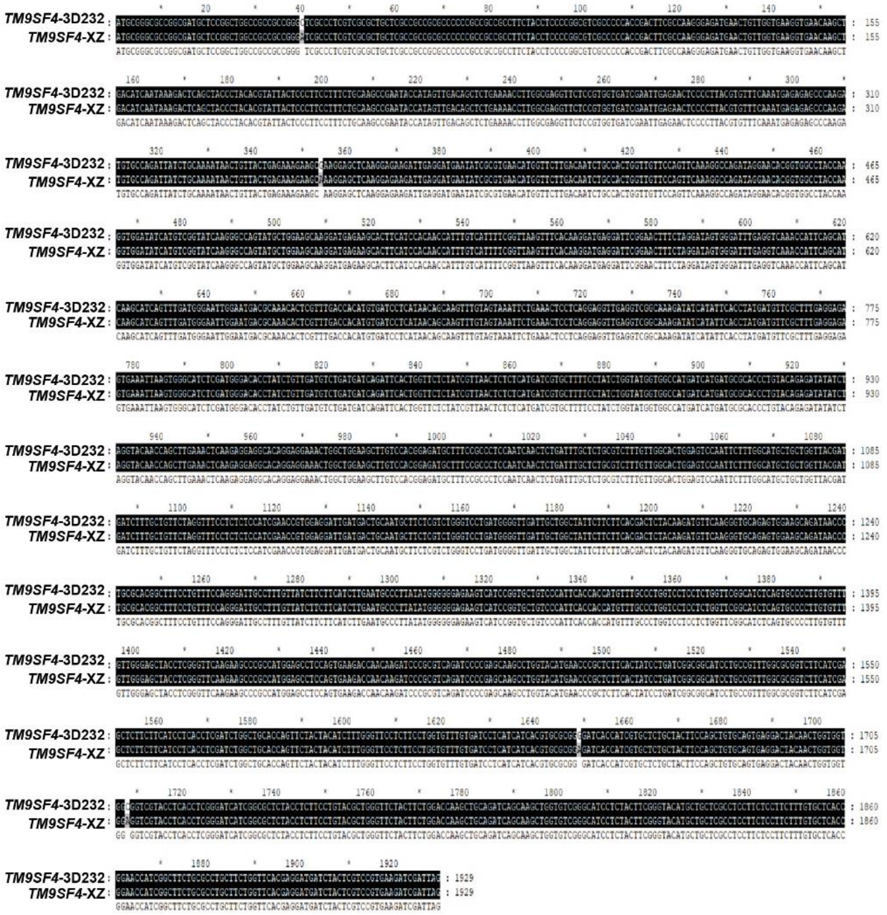

Supplementary Fig. 5. Coding sequence alignment of *TM9SF4* gene from lines 3D232 and Xuezao (XZ).

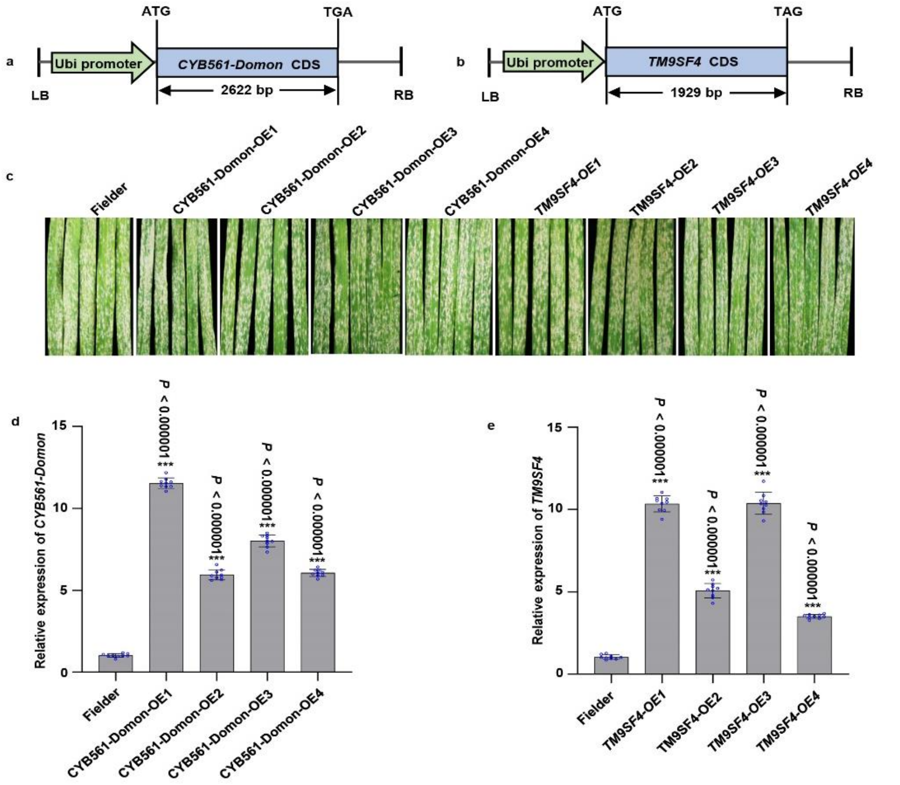

Supplementary Fig. 6. Validation of *CYB561-Domon* and *TM9SF4* by transgenic assay. a, b Structure of *ProUbi*: *CYB561-Domon* (a) and *ProUbi*: *TM9SF4* (b) construct used for transgenic assays, respectively. The *ProUbi*: *CYB561-Domon* construct contains the full length CDS of *CYB561-Domon* gene. The *ProUbi*: *TM9SF4* construct contains the full length CDS of *TM9SF4*. *Ubi*, promoter of the maize polyubiquitin gene. LB, left border; RB, right border. **c** Powdery mildew resistance assessments of *CYB561-Domon* and *TM9SF4* T_1 positive transgenic plants. Fielder, Xuezao, 3D232 and the T_1 positive transgenic plants of *ProUbi: CYB561-Domon* and *ProUbi: TM9SF4* were inoculated with *Bgt* isolate E09 at two-leaf stage. Representative leaves were photographed at 10 dpi. Three independent experiments were performed. **d, e** Relative transcript levels of *CYB561-Domon* and *TM9SF4* genes were examined by quantitative reverse transcription PCR (qRT-PCR). Leaves of seedling plants at the two-leaf stage were collected. *TaActin* was used as an endogenous control. Data are means ± SD from three biological replicates (three leaves used per biological replicate) and three technical replicates for each leave (n=9) were performed. The asterisks represent significance of differences by multiple unpaired *t* tests (**: $P < 0.01$, and ***: $P < 0.001$). Error bars are standard errors of the mean. Source data are provided as a Source Data file.

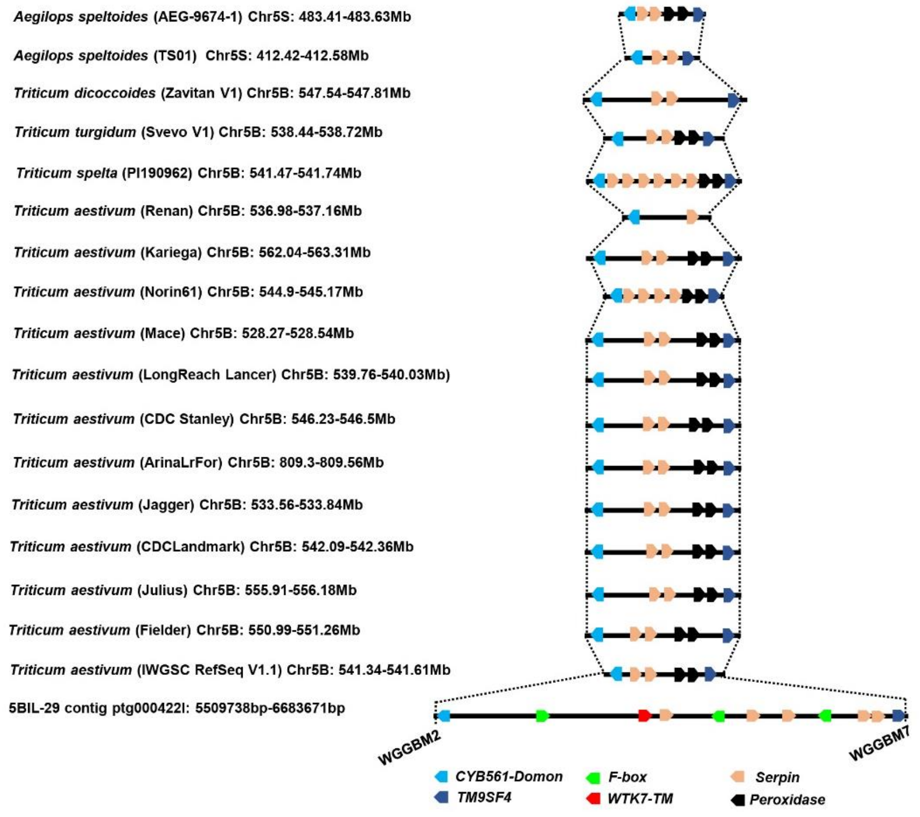

Supplementary Fig. 7. Collinearity analysis of *Ml3D232/ Pm36* locus in multiple *Triticeae* reference genomes. The closest flanking markers *WGGBM2* and *WGGBM7* of *Ml3D232/ Pm36* locus were used to determine the physical interval in multiple *Triticeae* reference genomes. The tetraploid WEW-durum introgression line 5BIL-29 carrying *Pm36* was sequenced through the PacBio SMRT long-read sequencing approach. A 7.1 Mb contig ptg000422l spanning the entire *Pm36* physical mapping interval was captured by aligning the flanking and co-segregating markers *WGGBM2* to *WGGBM7* around the *Pm36* locus. Eleven genes were annotated in the 1.17 Mb physical interval of *Pm36*: *CYB561-Domon* protein, three *F-box* proteins, five *Serpin* proteins, a tandem kinase protein with a single transmembrane domain in the C-terminus (*WTK7-TM*), and *TM9SF4*.

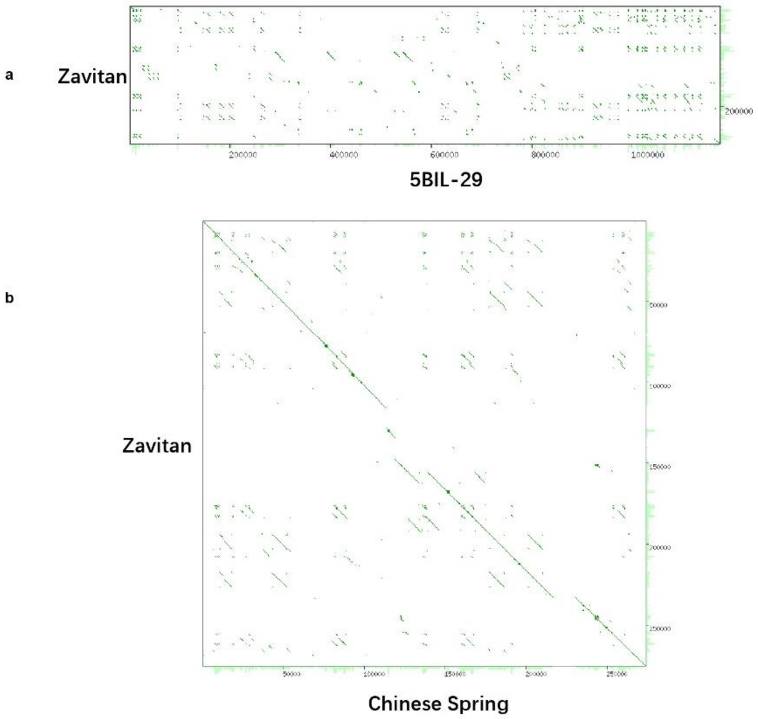

Supplementary Fig. 8. Genomic similarity comparison in *Ml3D232*/*Pm36* locus. The genomic sequences were cut out using the flanking markers *WGGBM2* and *WGGBM7* on the *Ml3D232*/*Pm36* locus based on Chinese Spring RefSeqv1.0 reference genome, Zavitan WEW_v1.0 reference genome and 5BIL-29 contig ptg000422l: 5509738-6683671. Sequence similarity comparisons were performed using YASS genomic similarity search tool (https://bioinfo.cristal.univ-lille.fr/yass/yass.php). **a** Zavitan (vertical) vs 5BIL-29 (horizontal); **b** Zavitan (vertical) vs Chinese Spring (horizontal).

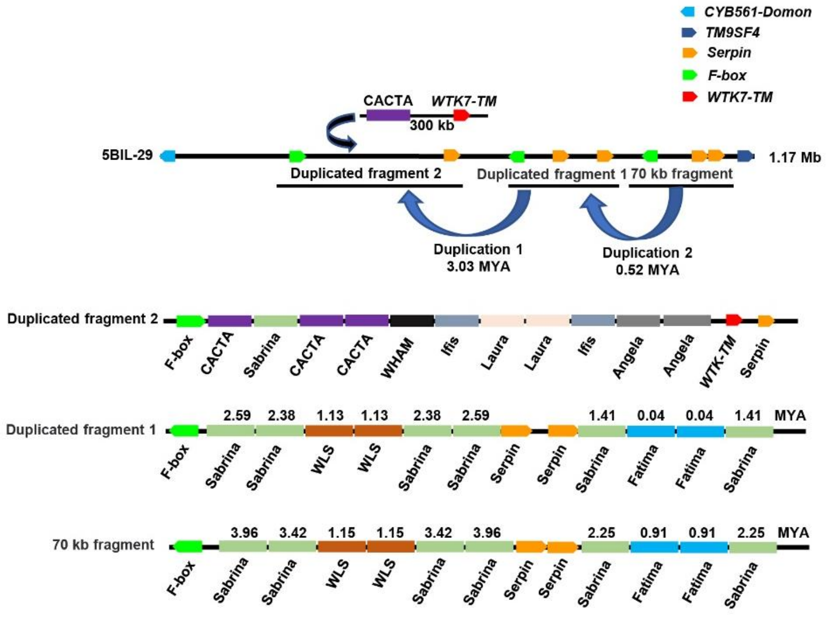

Supplementary Fig. 9. Schematic diagram of genomic evolution in *Ml3D232*/*Pm36* locus. Transposable elements (TEs) were identified through BLASTN against the *Triticeae* repetitive elements (TREP) (https://trep-db.uzh.ch/) using 5BIL-29 genomic sequence (contig ptg000422l: 5509738-6683671) between the flanking markers *WGGBM2* and *WGGBM7* in the *Ml3D232*/*Pm36* locus. The long terminal repeat sequences of LTR retrotransposable elements were delineated using Dotter analysis and manually checked. The evolutionary distance of the two LTR sequences was estimated by MEGA11 (https://www.megasoftware.net/) using a substitution rate of 1.3×10^{-8} mutations per site per year. An about 70 kb fragment containing one *F-box* and two *Serpin* genes was likely duplicated twice in the *Pm36* physical interval. Based on the sequences alignment of LTRs, the divergence time of Duplication 1 and Duplication 2 was estimated 0.52 million years ago (MYA) and 3.03 MYA, respectively. The insertion time of 10 intact LTRs were from 0.04 to 3.96 MYA, which indicated that this *Pm36* region was active for the gene/fragment duplication and LTRs played a leading role for this process. The tandem kinase *WTK7-TM* was identified in the 300 kb new sequences here.

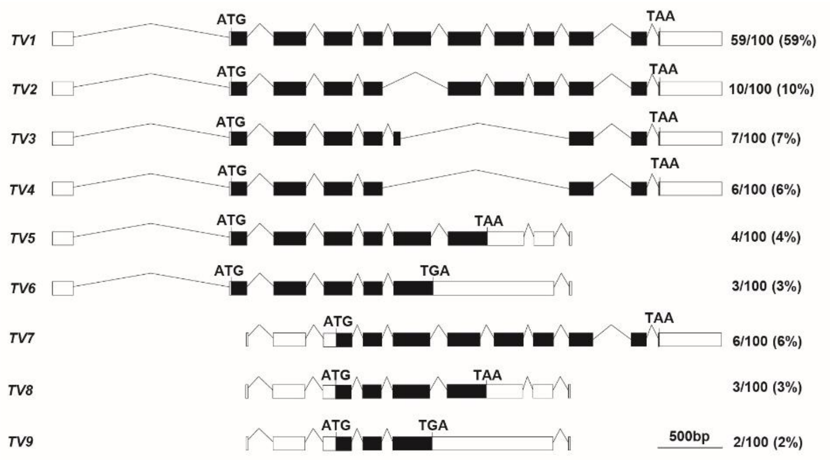

Supplementary Fig. 10. Transcript variants of *WTK7-TM*. Cloning and sequencing of the *WTK7-TM* cDNA clones identified nine transcript variants, designated *TV1* to *TV9* from line 3D232. One hundred colonies were sequenced using the Sanger sequencing. White and black boxes indicate portions of mature mRNA. Black boxes represent protein-coding region of the *WTK7-TM* cDNA. The caret-shaped lines indicate regions that are absent in mature mRNA aligned with genomic sequence of *WTK7-TM*. The numbers and percentages of variants are shown on the right, e.g., 59/100 (59%).

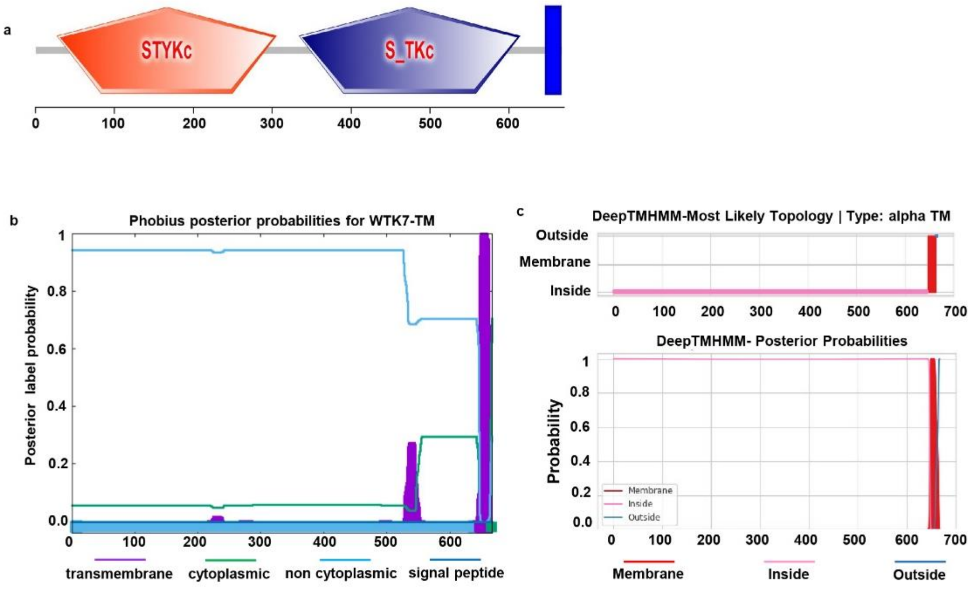

Supplementary Fig. 11. Prediction of protein transmembrane structure of WTK7-TM. a The domain architectures of WTK7-TM protein were predicted by SMART (https://smart.embl.de/). b Prediction of transmembrane helices was performed with Phobious (https://phobius.sbc.su.se/). **c** Transmembrane helices predicted with TMHMM server v.2.0 (http://www.cbs.dtu.dk/services/TMHMM/).

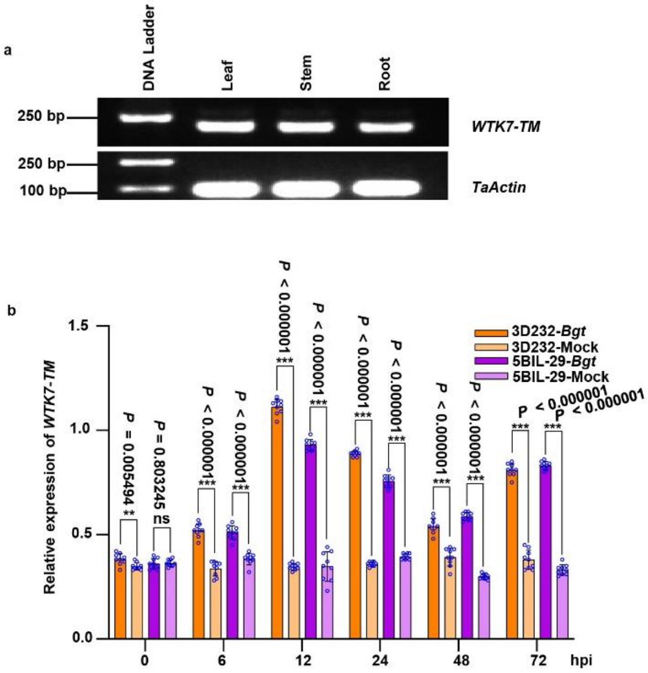

Supplementary Fig. 12. Expression pattern of *WTK7-TM* in line 3D232 and 5BIL-29. a The expression of *WTK7-TM* was detected in leaf, stem, and root of 3D232 by RT-PCR. *TaActin* was used as the endogenous control. Three biological replicates (three leaves or root or stem used per biological replicate) and three technical replicates for each sample were performed. **b** Relative transcript levels of *WTK7-TM* gene were examined by quantitative reverse transcription PCR (qRT-PCR). Leaves of 3D232 and 5BIL-29 seedling plants inoculated with *Bgt* isolate E09 at the two-leaf stage were collected at 0, 6, 12, 24, 48, and 72 hour post inoculation (hpi). *TaActin* was used as an endogenous control. Data are means ± SD from three biological replicates (three leaves used per biological replicate) and three technical replicates for each leave were performed. The asterisks represent significance of differences by multiple unpaired *t* tests (ns: not significant, **: $P < 0.01$, and ***: $P < 0.001$). Error bars are standard errors of the mean. Source data are provided as a Source Data file.

a

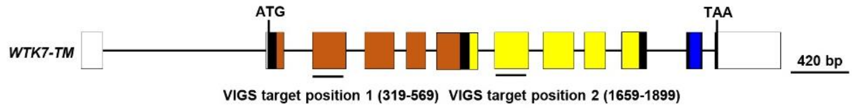

b

>VIGS1 target sequence:
GGGAATATTGGGAGATGGGCAAGAGATAGCTGTGAAGAAACTTTATCCCCAACTGTTCGATGGTGTGAGATTACAACAACAATA
TAATGAACTCATAGATATCAACCATGAAAATATTGTACGGTTAGTTAGCTGCTGCCAAGAGACACGATATAAGCATATTGAGCAC
AAGGGAGAGCTTGTTTTCGCCAAGCTGCAGGAAATTGCTCTTTGCTCGGAATACTTGCCAGGTGGAAGCCTTGACAAGCATG

>VIGS2 target sequence:
ACTTGATGATGGGGAAGAGATTGCTGTGAAGAAGCTTAAAAGCATGCCGCTGCTTGATGACCATCAATTTAATAATGAGTTAAAC
AATCTTAAGACACTTGAGCATCCAAATATTGTACGGTTAGTTGGCTACTGCCATGATACGACACTAGTAAATGTTGAGCACAATG
GAAGAGTGGTTTGTGCTGCTGTGGAGGAAAGAGCTTTATGCTACGAATACTTGGAGGAAGGAAGCCTTGAC

Supplementary Fig. 13. The target positions and sequences of *WTK7-TM* in BSMV-VIGS experiments. a Two target positions of *WTK7-TM* located on domains Kin I and Kin II, respectively, in the VIGS assay indicated by black transverse line. **b** Target sequences of *WTK7-TM* in the VIGS assay.

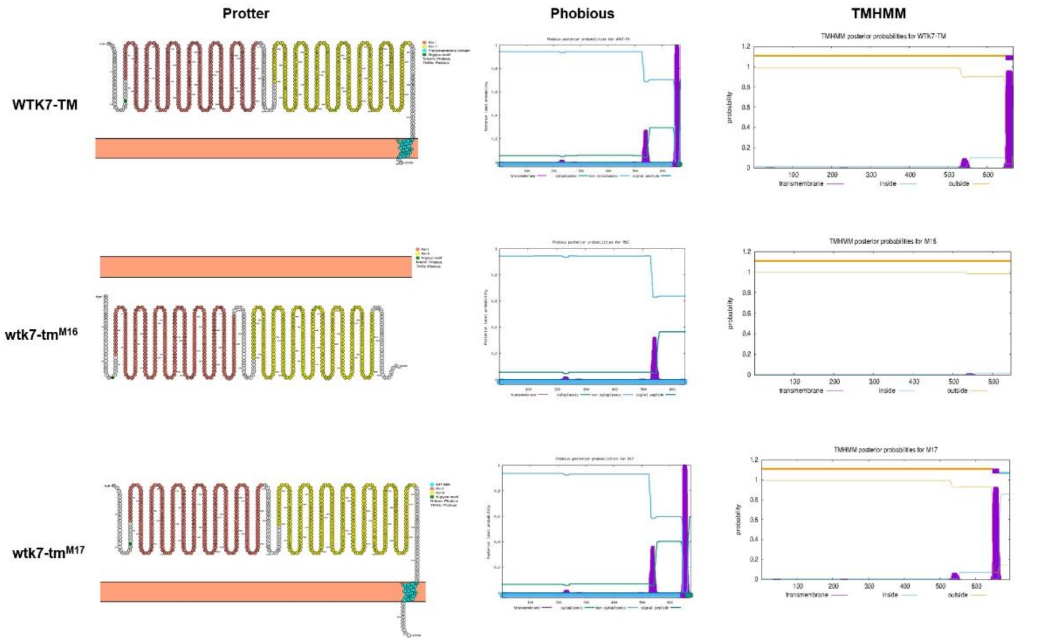

Supplementary Fig. 14. Protein topology prediction of WTK7-TM and wtk7-tm. The WTK7-TM, wtk7-tm^{M16} (EMS mutant M16) and wtk7-tm^{M17} (EMS mutant M17) were used to predict the protein topology in Protter website (http://wlab.ethz.ch/protter/#), Phobious (https://phobius.sbc.su.se/), TMHMM server v.2.0 (http://www.cbs.dtu.dk/services/TMHMM/). Kinase I (Kin I), kinase II (Kin II) and transmembrane (TM) domains of tandem kinase proteins are highlighted in orange, yellow and cyan circle, respectively.

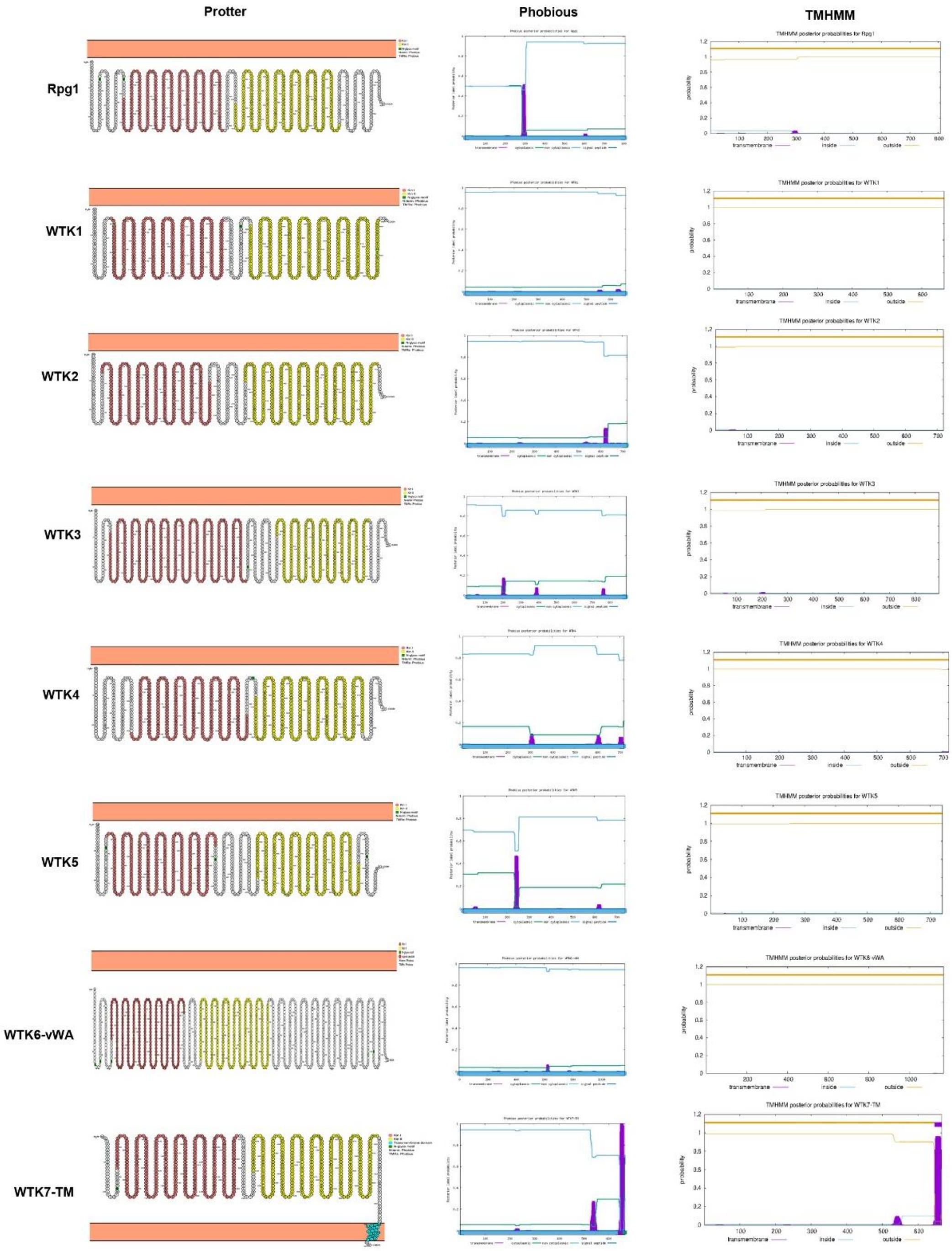

Supplementary Fig. 15. Protein topology prediction of reported tandem kinase proteins in wheat and barley. The reported tandem kinase proteins, Rpg1, WTK1, WTK2, WTK3, WTK4, WTK5, WTK6-vWA and WTK7-TM were used to predict the protein topology in Protter website (http://wlab.ethz.ch/protter/#), Phobious (https://phobius.sbc.su.se/), TMHMM server v.2.0 (http://www.cbs.dtu.dk/services/TMHMM/). Kinase I (Kin I), kinase II (Kin II) and transmembrane (TM) domains of tandem kinase proteins are highlighted in orange, yellow and cyan circle, respectively.

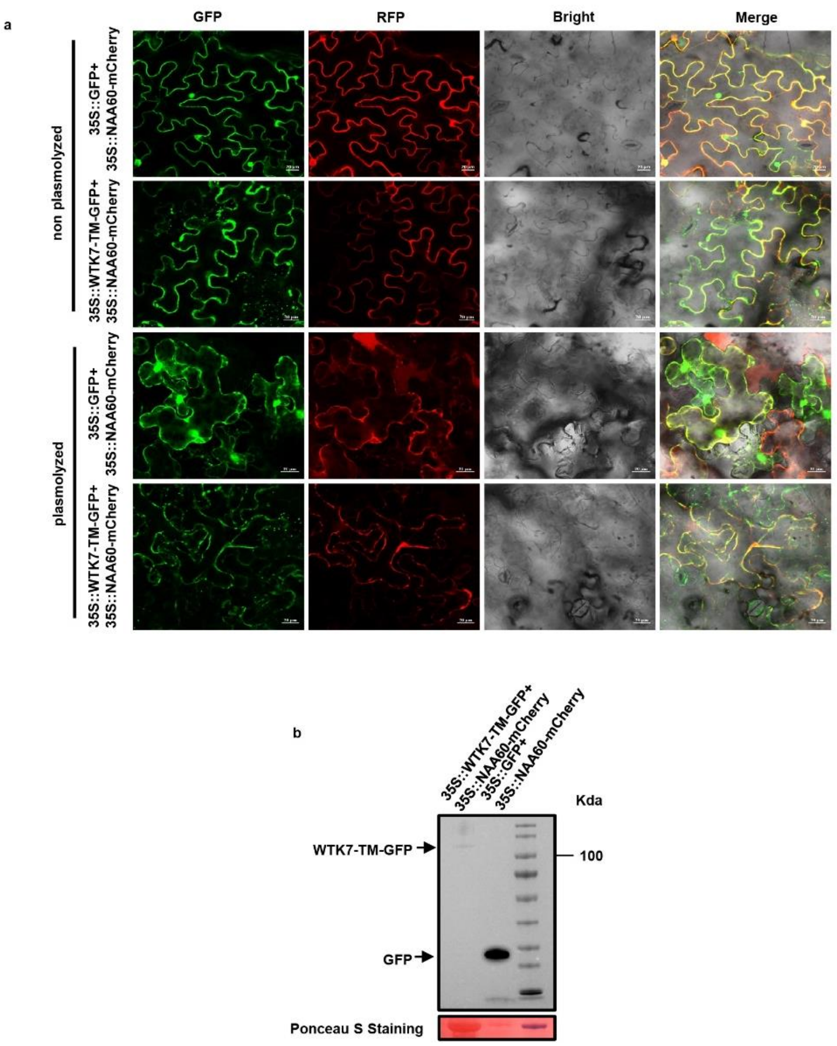

Supplementary Fig. 16. Subcellular localization of WTK7-TM. a Subcellular localization of WTK7-TM in *Nicotiana benthamiana* leaf epidermal cells. 35S:WTK7-TM-GFP+35S:NAA60-mCherry or 35S:GFP+35S:NAA60-mCherry fusion proteins were expressed in *N. benthamiana* leaves through agroinfiltration. NAA60 is a plasma membrane marker protein. To induce plasmolysis, leaf samples were incubated 10-15 min in 4% NaCl before imaging. The confocal microscopy images were captured 2 dpi using a confocal laser scanning microscope (Carl Zeiss, LSM880). Scale bars, 20 μm. Three independent experiments were performed. **b** Protein extracts prepared from the infiltrated leaves were analyzed by immunoblotting with anti-GFP antibody. Three independent experiments were performed. Source data are provided as a Source Data file.

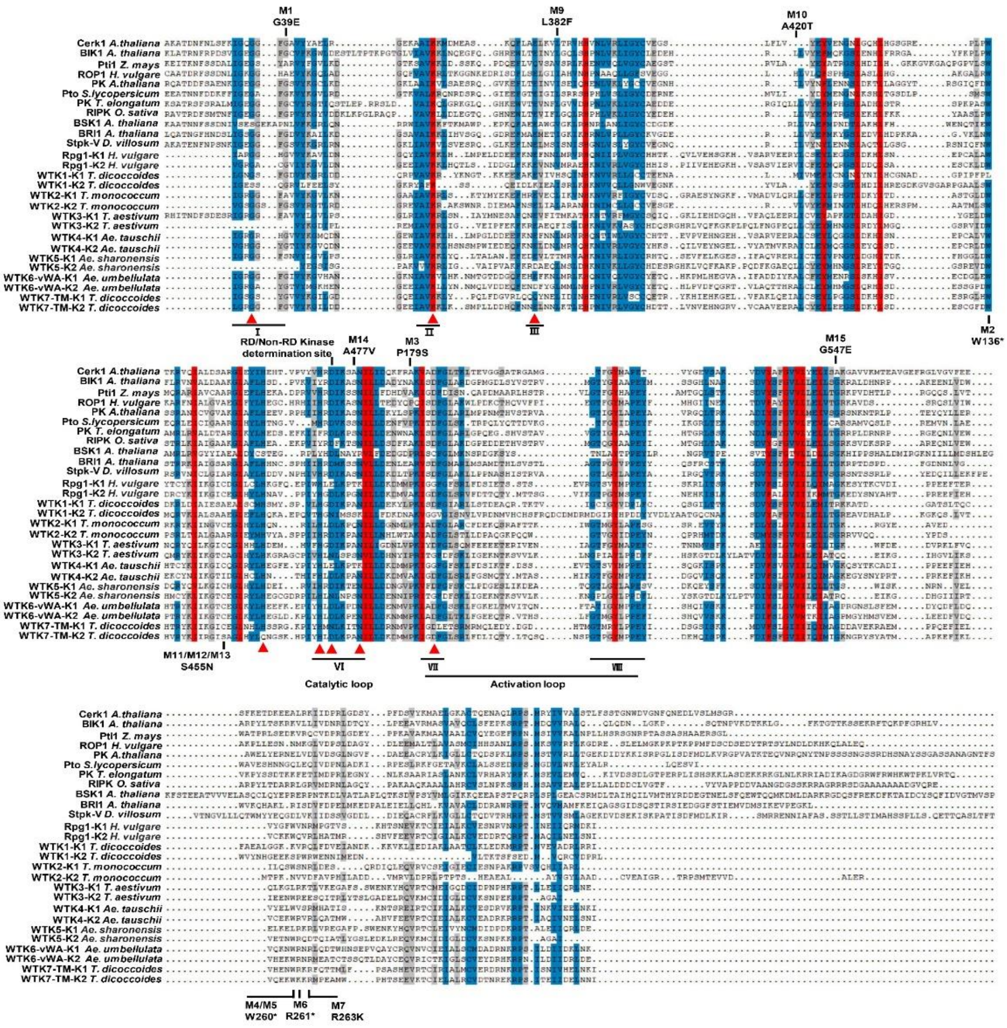

Supplementary Fig. 17. Multiple alignment of protein kinase domains. Twenty-five reported protein kinase domains were used to infer the architecture of WTK7-TM. Cerk1 *Arabidopsis thaliana* (NP_566689.2), BIK1 *A. thaliana* (OPA11046.1), Pti1 *Zea mays* (NP_001105753.2),ROP1 *Hordeum vulgare* (CCE57823.1), PK *A. thaliana* (NP_564003.1), Pto *Solanum lycopersicum* (XP_025886705.1), PK *Thinopyrum elongatum* (AAK11674.1), RIPK *Oryza sativa* (XP_015630876.1), BSK1 *A. thaliana* (OAO99810.1), BRI1 *A. thaliana* (AAC49810.1), Stpk-V *Dasypirum villosum* (AEF30547.1), Rpg1-K1 *H. vulgare* (AAM76922.1), Rpg1-K2 *H. vulgare* (AAM76922.1), WTK1-K1 *T. dicoccoides* (MG649384), WTK1-K2 *T. dicoccoides* (MG649384), WTK2-K1 *T. monococcum* (MK629715.1), WTK2-K2 *T. monococcum* (MK629715.1), WTK3-K1 *T. aestivum* (MK953855), WTK3-K2 *T. aestivum* (MK953855), WTK4-K1 *Aegilops tauschii* (MW295405.1), WTK4-K2 *Ae. tauschii* (MW295405.1), WTK5-K1 *Ae. sharonensis* (MZ826707.1), WTK5-K2 *Ae. Sharonensis* (MZ826707.1), WTK6-vWA-K1 *Ae. umbellulate* (WDY61369.1), WTK6-vWA-K2 *Ae. umbellulate* (WDY61369.1), WTK7-TM-K1 *T. dicoccoides,* and WTK7-TM-K2 *T. dicoccoides.* Key conserved residues indicated by red triangles. Amino acids altered by EMS mutagenesis are indicated by black lines.

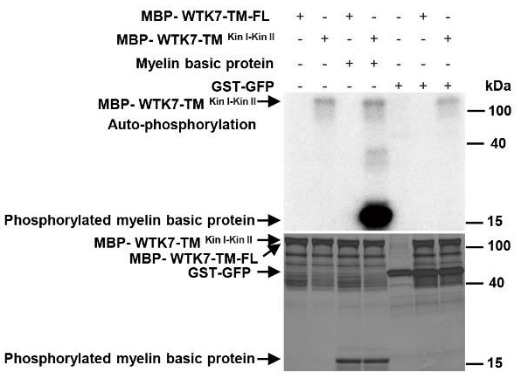

Supplementary Fig. 18. Kinase activity analysis of full length WTK7-TM in vitro. Compared to the truncated version MBP-WTK7-TM$^{\text{Kin I-Kin II}}$ composing of Kin I+ Kin II without transmembrane of WTK7-TM (1-646 aa), the full length WTK7-TM (1-667 aa) lacks kinase activity. Autoradiograph and coomassie brilliant blue staining are shown in the top and bottom panels, respectively. As a negative control, GST-GFP was used as substrate. FL, full length. Three independent experiments were performed. Source data are provided as a Source Data file.

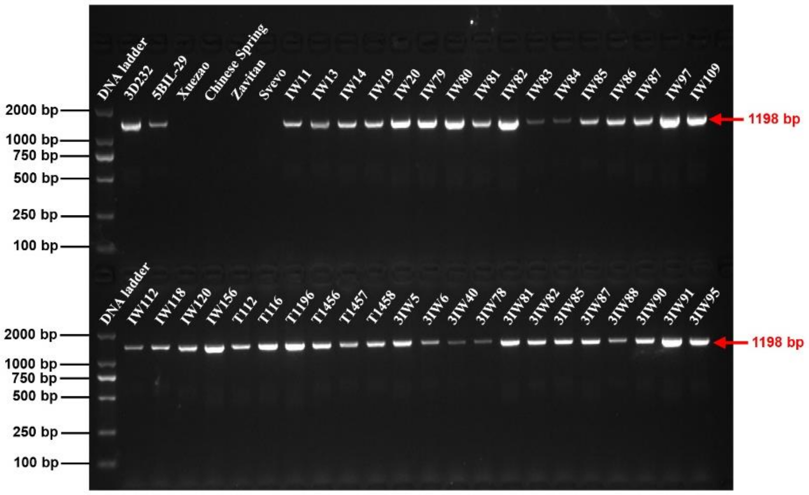

Supplementary Fig. 19. Development and validation of the functional marker for *WTK7-TM*. Based on the genomic sequence of *WTK7-TM* gene, we developed a specific primer, *WTK7-TM-FM*, to identify the presence of the *WTK7-TM* functional allele. The dominant functional marker *WTK7-TM-FM* amplified a 1,198 bp PCR product if the plant carrying *WTK7-TM* gene, otherwise no amplification product. Three independent experiments were performed. Source data are provided as a Source Data file.

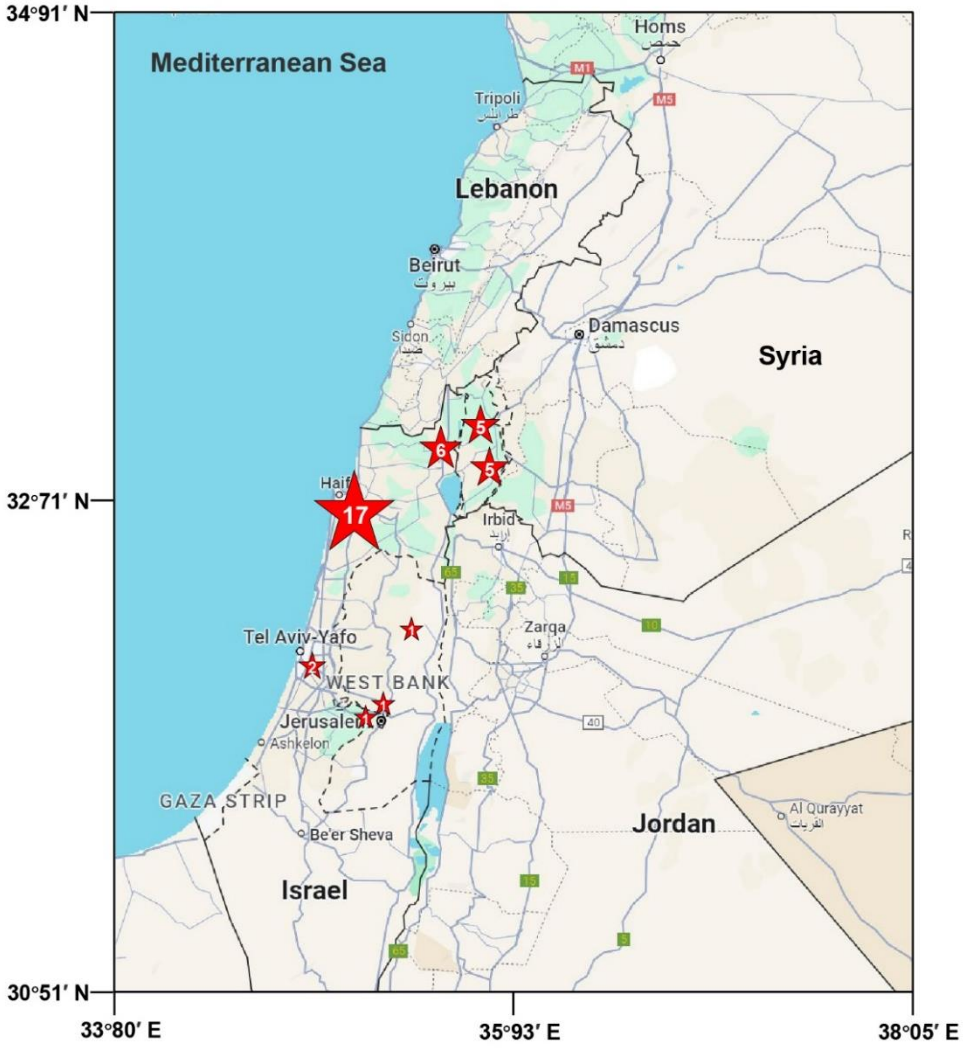

Supplementary Fig. 20. Geographic distribution of *WTK7-TM* in wild emmer wheat natural populations. Red stars represent the location of 38 wild emmer wheat carrying *WTK7-TM*. Numbers in red stars indicate the amount of wild emmer wheat carrying *WTK7-TM*. This map was made using geographic data from Google Maps. N: North latitude; E: East longitude.
