# Supplementary material for: A membrane associated tandem kinase from wild emmer wheat confers broad-spectrum resistance to powdery mildew
Source: Nat Commun. 2024 Apr 10;15:3124. doi: 10.1038/s41467-024-47497-w (PMC11006675; doi:10.1038/s41467-024-47497-w)
Supplement: Supplementary file 3 — Description of Additional Supplementary Files [file 41467_2024_47497_MOESM3_ESM.pdf]

Description of Additional Supplementary Files

File Name: Supplementary Data 1 Description: Infection type of line 3D232 and Chancellor to divergent *Bgt* isolates.
File Name: Supplementary Data 2 Description: Primers used in the study.
File Name: Supplementary Data 3 Description: Statistics of the WEW-durum introgression line 5BIL-29 genome assembly.
File Name: Supplementary Data 4 Description: Compared physical length at the *Ml3D232*/*Pm36* locus among different genomes.
File Name: Supplementary Data 5 Description: RNA-seq analysis of annotated genes at the Pm36 locus in line 5BIL-29 inoculated with *Bgt* isolate E09.
File Name: Supplementary Data 6 Description: Molecular characterization of the *WTK7-TM* EMS mutants.
File Name: Supplementary Data 7 Description: Distribution of *WTK7-TM* in tetraploid and hexaploid wheat natural populations.
File Name: Supplementary Data 8 Description: Infection types (ITs) of WEW accessions carrying *WTK7-TM* to *Bgt* isolates E09 and E20.
